# The Combined Effects of Hydraulic Calcium Silicate Cement and Enamel Matrix Derivative Regarding Osteogenic and Dentinogenic Differentiation on Human Dental Pulp Stem Cells

**DOI:** 10.3390/ma16114003

**Published:** 2023-05-26

**Authors:** Ji-Young Yune, Donghee Lee, Sin-Young Kim

**Affiliations:** 1 Graduate School of Clinical Dental Science, The Catholic University of Korea, 222 Banpo-daero, Seoul 06591, Republic of Korea; joannayune@hanmail.net; 2 Department of Dentistry, College of Medicine, The Catholic University of Korea, 222 Banpo-daero, Seoul 06591, Republic of Korea; dong524@catholic.ac.kr; 3 Department of Conservative Dentistry, Seoul St. Mary’s Hospital, College of Medicine, The Catholic University of Korea, 222 Banpo-daero, Seoul 06591, Republic of Korea

**Keywords:** hydraulic calcium silicate cement, enamel matrix derivative, mineral trioxide aggregate, human dental pulp stem cell, osteogenic potential, dentinogenic potential

## Abstract

The ideal treatment option for immature necrotic permanent teeth is regeneration of the pulp–dentin complex. Mineral trioxide aggregate (MTA), the conventional cement used for regenerative endodontic procedures, induces hard tissue repair. Various hydraulic calcium silicate cements (HCSCs) and enamel matrix derivative (EMD) also promote osteoblast proliferation. The purpose of the present study was to determine the osteogenic and dentinogenic potential of commercially distributed MTA and HCSCs when applied in combination with Emdogain gel on human dental pulp stem cells (hDPSCs). The presence of Emdogain resulted in greater cell viability, and higher alkaline phosphatase activity was detected in the Emdogain-supplemented groups in the early days of cell culture. On qRT–PCR, the groups treated, respectively, with Biodentine and Endocem MTA Premixed in the presence of Emdogain showed an increased expression of the dentin formation marker *DSPP*, and the group treated with Endocem MTA Premixed in the presence of Emdogain showed an upregulated expression of the bone formation markers *OSX* and *RUNX2*. In an Alizarin Red-S staining assay, all of the experimental groups exhibited a greater formation of calcium nodules when treated in combination with Emdogain. Overall, the cytotoxicity and osteogenic/odontogenic potential of HCSCs were similar to that of ProRoot MTA. The addition of the EMD increased the osteogenic and dentinogenic differentiation markers.

## 1. Introduction

A regenerative endodontic procedure is defined as a biology-based technique that replaces damaged or absent dentin and root structures by incorporating cells from the pulp–dentin complex. The importance of pulp–dentin complex regeneration is emphasized in immature permanent teeth with pulp necrosis due to infection from dental caries, trauma, and tooth fractures [1]. The traditional therapies for non-vital immature permanent teeth consist of apexification, revascularization, or regenerative endodontic procedures [2]. Mineral trioxide aggregate (MTA) is widely used in pulp therapy, with a crucial role in immature permanent teeth in particular. MTA has excellent biocompatibility, sealing ability, and antibacterial effects [3]. The earliest released product was ProRoot MTA (Dentsply Tulsa Dental Specialties, Tulsa, OK, USA), which enhances the gene expression markers involved in the bone, dentin, and vascular formation of human dental pulp stem cells (hDPSCs) [4]. However, ProRoot MTA has several disadvantages, such as an extended setting time, difficulty in manipulation, and discoloration [5,6]. The newly introduced hydraulic calcium silicate cements (HCSCs) overcome these shortcomings and have been deployed successfully on a commercial scale in local dental clinics [7]. Among such HCSCs, Biodentine (Septodont, Saint-Maur-des-Fosses, France) has shown good results in both animal trials and clinical trials [8,9,10], and Endocem MTA Premixed (Maruchi, Wonju, Republic of Korea) is packaged in a syringe for easy operability. Despite the clinical advantage of easy application, continuous research is required to supplement long-term data and assess potentially enhancing additional treatments. Enamel matrix derivative (EMD) is constituted from amelogenin, a hydrophobic protein that accounts for 90% of enamel protein. Because it does not dissolve at physiological pH or body temperature, EMD is less likely to act as an antigen and can instead act as an important stimulator of acellular cementum formation [11,12,13,14]. Recently, in the field of endodontics, EMD application at the external root apex has been reported to induce formation of cementum-like tissue, and application at the exposed dental pulp can be used to induce the formation of dentin-like hard tissue [15,16,17,18] The purpose of the present study was to analyze the osteogenic and dentinogenic potential of ProRoot MTA, Biodentine, and Endocem MTA Premixed when applied on hDPSCs in combination with Emdogain gel (Straumann, Basel, Switzerland), a commercially available EMD.

## 2. Materials and Methods

### 2.1. Human Dental Pulp Stem Cells (hDPSCs)

This research was granted approval by the Institutional Review Board at the Catholic University of Korea (IRB No. MC21ZAS10062). To conduct this study, we obtained fourth-passage hDPSCs from Top Cell Bio, Inc. (Seoul, Republic of Korea), while maintaining source anonymity. The cells were maintained in a growth medium (GM) comprising HyClone Minimum Essential Medium (α-MEM; Cytiva, Marlborough, MA, USA) supplemented with HyClone 10% fetal bovine serum (Cytiva), 100 U/mL penicillin, and 100 μg/mL streptomycin.

The osteogenic/dentinogenic medium (OM) was formulated by combining specific components, including HyClone Minimum Essential Medium (α-MEM), 50 μg/mL ascorbic acid (Sigma-Aldrich, St. Louis, MO, USA), 0.1 µM dexamethasone (Sigma-Aldrich), and 10 mM beta-glycerophosphate (Sigma-Aldrich). The experiments were conducted under aseptic conditions at 37 °C in the presence of 5% CO_2._ This medium is typically used to assess the osteogenic/odontogenic potential of cells.

### 2.2. Production of Experimental MTA, HCSC Disks, or Eluates

We used the conventional ProRoot MTA, HCSCs such as Biodentine, and Endocem MTA Premixed. The composition of each material is listed in Table 1. Under sterilized conditions, the manufacturer’s instructions were followed to mix each cement. A sterilized rubber mold was used to set the disk-shaped specimens. Each specimen was standardized to a height of 3 mm and diameter of 6 mm. The specimen disks were placed under wet gauze at room temperature for 72 h. After they had set, all disks were subjected to ultraviolet sterilization for 4 h. Two disks from each experimental group were eluted in 40 mL of cell culture medium in a 37 °C incubator for 7 days. A 0.2-μm pore filter (Minisarts CA Syringe Filters; Sartorius, Goettingen, Germany) was used for filtration, and each conditioned medium was diluted with the cell culture medium to achieve a final concentration of 5 mg/mL. The concentration of the eluates was determined according to our pilot study.

### 2.3. Classification of the Groups

Eight experimental groups were established based on combinations of controls and treatments both with and without Emdogain (30 mg/mL; added at a concentration of 100 μg/mL by dilution), as follows: ProRoot MTA disks or eluates, manufactured as described above, with or without Emdogain gel added; Biodentine disks or eluates, also manufactured as described above, with or without Emdogain; Endocem MTA Premixed disks or eluates, with or without Emdogain; and controls made without the experimental disks or eluates, with or without Emdogain.

### 2.4. Cell Toxicity Measurement

The cytotoxic effect of the experimental disks on the hDPSCs was evaluated with the 3-(4,5-dimethylthiazol-2-yl)-2,5-diphenyl tetrazolium bromide (MTT) assay. The color change from the reaction between the MTT solution and the dehydrogenase in cell mitochondria was analyzed on Days 2, 4, and 6. The hDPSCs were cultured in GM at a density of 1.0 × 10^4^ cells/well in 24-well cell culture plates (SPL Life Sciences, Pocheon, Republic of Korea). Individual disks were placed on an insert (SPLInsert; SPL Life Sciences) above the hDPSCs. As a control group, no experimental disks were used, and only hDPSCs were cultured in GM. For each well, 0.5 mg/mL of the MTT solution was added and incubated at 37 °C for 4 h. When purple-colored formazan was observable, crystals were solubilized with 200 μm of dimethyl sulfoxide by gently shaking for 30 min. Absorption at 570 nm was measured using a microplate reader, with six independent experiments for each group.

### 2.5. Live/Dead Staining Assay

Live cells consist of an intact plasma membrane with active esterase activity between cells. Green-fluorescent calcein-AM staining representing esterase activity was observed using a fluorescence microscope on Days 2 and 6. The hDPSCs were cultured in GM at a density of 1.0 × 10^4^ cells/well in 24-well cell culture plates. After 24 h of incubation, each experimental disk was released above the hDPSCs and incubated for 6 days. The growth medium was exchanged every 2 days. Using the LIVE/DEAD Cell Imaging Kit (488/570; Thermo Fisher Scientific, Waltham, MA, USA), green-fluorescent calcein-AM was used to detect the esterase activity between cells, and red-fluorescent ethidium homodimer-1 was used to distinguish the loss of the plasma membrane. Staining was carried out on Days 2 and 6 and observed using an inverted microscope (Axiovert 200; Carl Zeiss Microscopy, Jena, Germany). We performed quantitative analyses of cell viability using ImageJ 1.46r software (National Institutes of Health, Bethesda, MD, USA). Each group was measured in three independent experiments.

### 2.6. Alkaline Phosphatase (ALP) Activity

The ALP activity was evaluated on Days 2, 4, and 6. The hDPSCs were cultured in OM at a density of 0.7 × 10^4^ cells/well. Individual disks were released above the hDPSCs using an insert (SPLInsert). The control group consisted of hDPSCs that were cultured in OM without experimental disks. To conduct the ALP analysis, 50 μL of Senso-Lyte^®^ p-nitrophenylphosphate from the alkaline phosphatase assay kit (AnaSpec, Fremont, CA, USA) was combined with the supernatant of each specimen and mixed with the reagent for 30 s. Incubation was carried out for 30 min at 4 °C, and the absorption was measured at 405 nm using a microplate reader. Each group was measured in six independent experiments.

### 2.7. Quantitative Real-Time Polymerase Chain Reaction (qRT–PCR)

To evaluate osteogenic/odontogenic marker expression, qRT–PCR was performed on Day 7. Over 7 days, hDPSCs were incubated using the respective experimental disk eluates. The hDPSCs cultured in OM without experimental eluates served as a control group. The RNeasy Mini Kit (Qiagen, Hilden, Germany) was used to extract the total RNA from each cell, and the RevertAid First Strand cDNA Synthesis Kit (Thermo Fisher Scientific, Waltham, MA, USA) was used to induce reverse transcription and synthesize cDNA. For this experiment, we designed primers specific for the bone-formation markers runt-related transcription factor 2 (*RUNX2*) and osterix (*OSX*), and for the odontogenic markers dentin matrix protein-1 (*DMP-1*) and dentin sialophosphoprotein (*DSPP*). The primers were designed in GenBank (Table 2). RNA isolation and qRT–PCR were performed according to our previous work [19]. Amplified gene levels were standardized to *GAPDH* expression and each evaluated by fold change compared with that of the control. Each group was measured in three independent experiments.

### 2.8. Alizarin Red-S (ARS) Staining Assay

To evaluate the calcified nodules formed from human dental stem cells, the ARS staining assay was performed on Days 7 and 14. The hDPSCs were inoculated at a density of 0.5 × 10^4^ cells/well. Over 14 days, hDPSCs were incubated using all experimental disk eluates. The hDPSCs cultured in OM without experimental eluates were used as the control group. The cells were fixed with 4% paraformaldehyde solution for 20 min and stained with 2% ARS solution (ScienCell, Carlsbad, CA, USA) on Days 7 and 14. To stain, 10% cetylpyridinium chromide (Sigma-Aldrich) was applied for 15 min. Each group was measured in six independent experiments.

### 2.9. Statistical Analysis

For the statistical analysis, we used SPSS (ver. 24.0; IBM Corp., Armonk, NY, USA). Shapiro–Wilk normality verification was used to evaluate the distribution of the data. One-way analysis of variance was applied, with Tukey’s post hoc test, to identify any differences among all of the experimental groups. Paired *t*-tests were used to identify differences between the groups based on the presence/absence of Emdogain.

## 3. Results

### 3.1. Cell Toxicity Measurement

**Figure 1:**
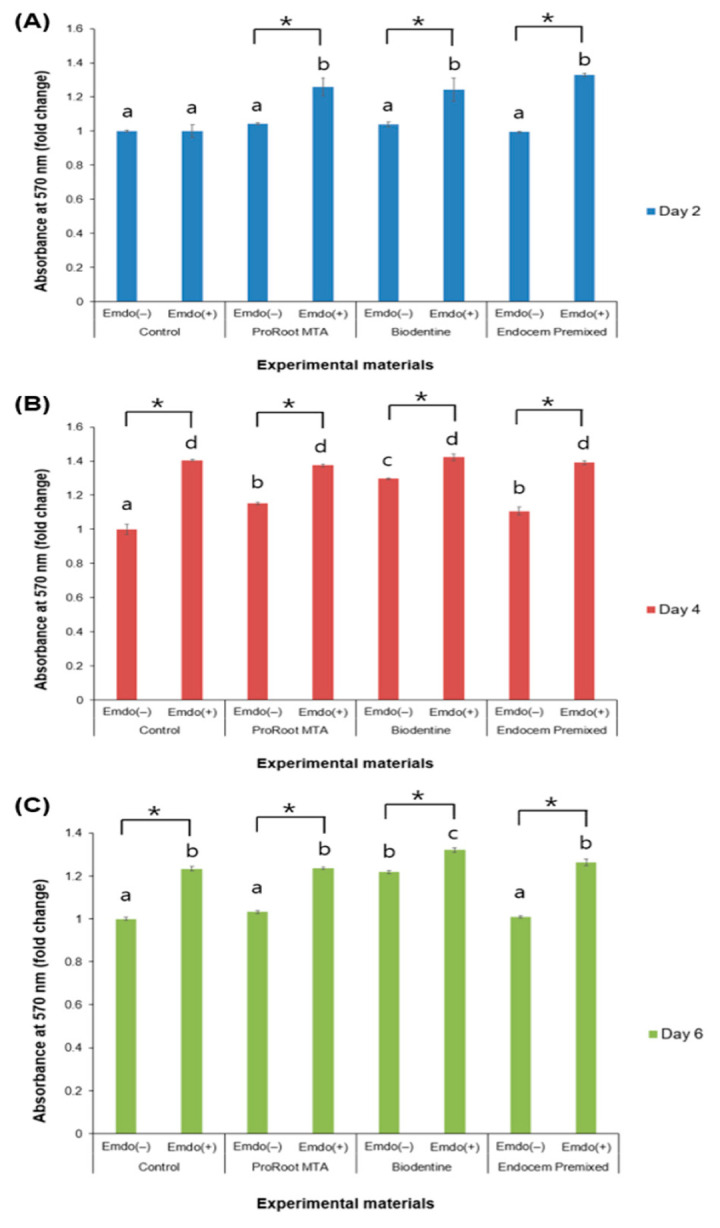
Comparison of cell viability according to the presence or absence of Emdogain gel (Emdo). (**A**) Day 2, (**B**) Day 4, and (**C**) Day 6. The different letters indicate significant differences between all groups (*p* < 0.05). The asterisks indicate significant differences between the presence and absence of Emdogain gel. The error bars indicate standard deviations.

In the absence of Emdogain gel, no significant difference was found among the experimental groups on Day 2 (Figure 1A). On Days 4 and 6, the Biodentine group displayed increased cell proliferation compared to the ProRoot MTA and Endocem MTA Premixed groups when Emdogain gel was not used (Figure 1B,C, *p* < 0.05). On Day 2, the experimental groups that were treated with Emdogain gel demonstrated a substantially higher level of cell viability compared to those that were not treated with Emdogain (Figure 1A, *p* < 0.05). Nonetheless, the control groups with and without Emdogain gel did not differ on Day 2. On Days 4 and 6, there was significant higher cell proliferation in Emdogain gel-treated experimental and control groups compared with the groups that were not treated with Emdogain gel (Figure 1B,C, *p* < 0.05).

### 3.2. Live/Dead Staining Analysis

**Figure 2:**
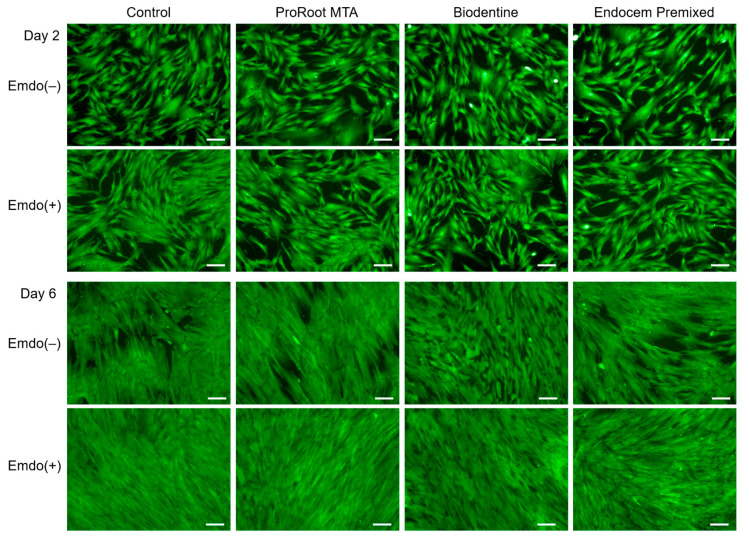
Representative images from live/dead staining analysis on Days 2 and 6. Scale bars = 200 µm.

**Figure 3:**
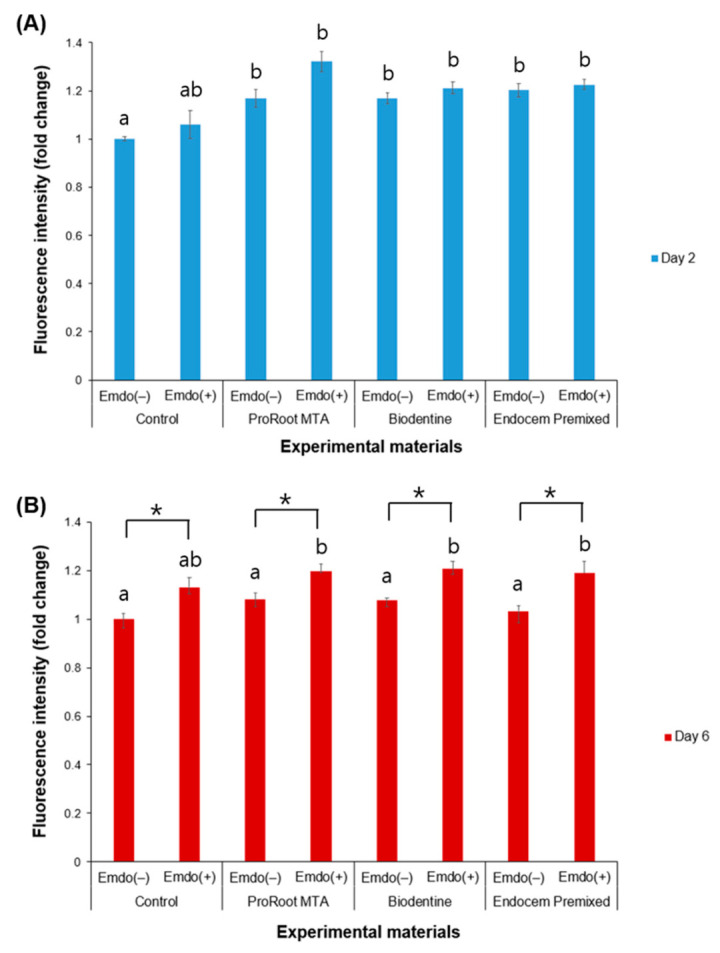
Comparison of live/dead staining analysis by the presence or absence of Emdogain gel (Emdo). (**A**) Day 2 and (**B**) Day 6. The different letters indicate significant differences between all groups (*p* < 0.05). The asterisks indicate significant differences between the presence and absence of Emdogain. The error bars indicate standard deviations.

Among the groups that were treated with Emdogain gel, the control and experimental groups did not differ (Figure 2), and the green-stained area generally increased on Day 6 compared to Day 2. The density of the green-stained area increased overall by Day 6 in the Emdogain-treated groups compared to the groups that were not treated with Emdogain (Figure 2). In the quantitative analysis, no significant differences were found on Day 2 among the experimental groups when Emdogain gel was not applied. We also found no significant difference among all of the groups, with or without Emdogain application, on Day 2. In contrast, on Day 6, the Emdogain-treated experimental and control groups had higher cell viability than the untreated groups (Figure 3, *p* < 0.05).

### 3.3. ALP Activity

**Figure 4:**
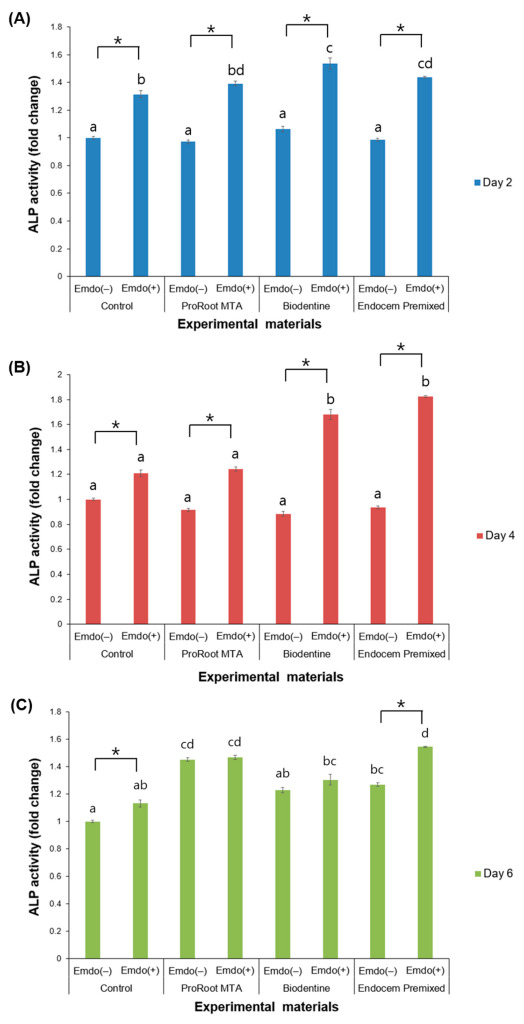
Comparison of ALP activity by the presence or absence of Emdogain gel (Emdo). (**A**) Day 2, (**B**) Day 4, and (**C**) Day 6. The different letters indicate significant differences between all groups (*p* < 0.05). The asterisks indicate significant differences between the presence and absence of Emdogain. The error bars indicate standard deviations.

When the Emdogain gel was not utilized, the experimental groups did not differ on Days 2 and 4 (Figure 4A,B). ProRoot MTA and Endocem MTA Premixed, both without Emdogain, had significantly higher ALP activities on Day 6 compared with the control group without Emdogain (Figure 4C, *p* < 0.05). When the Emdogain gel was applied, the control and all of the experimental groups had significantly higher ALP activities on Days 2 and 4 compared with those that were not treated with Emdogain (Figure 4A,B, *p* < 0.05). On Day 6, only the control and Endocem MTA Premixed groups had significantly higher ALP activities when the Emdogain gel was applied (Figure 4C, *p* < 0.05).

### 3.4. qRT–PCR

**Figure 5:**
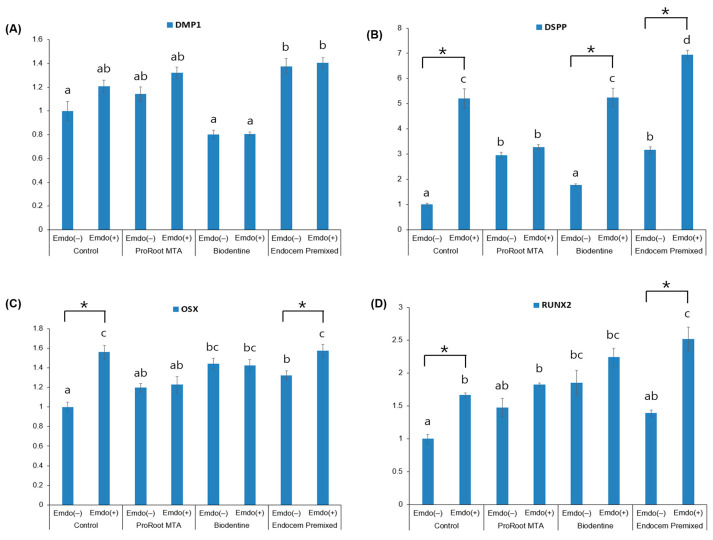
Comparison of osteogenic/odontogenic marker expression on Day 7 by the presence or absence of Emdogain gel (Emdo). (**A**) *DMP-1*, (**B**) *DSPP*, (**C**) *OSX,* and (**D**) *RUNX2*. The different letters indicate significant differences between all groups (*p* < 0.05). The asterisks indicate significant differences between Emdogain gel application and non-application. The error bars indicate standard deviations.

The expression of the osteogenic/odontogenic markers increased when the Emdogain gel was applied (Figure 5). In particular, *DSPP* expression significantly increased in the presence of Emdogain in the control, Biodentine, and Endocem MTA Premixed groups (Figure 5B, *p* < 0.05). The expression of *OSX* and *RUNX2* also significantly increased in the control and Endocem MTA Premixed groups when the Emdogain gel was used (Figure 5C,D, *p* < 0.05).

### 3.5. ARS Staining Assay

**Figure 6:**
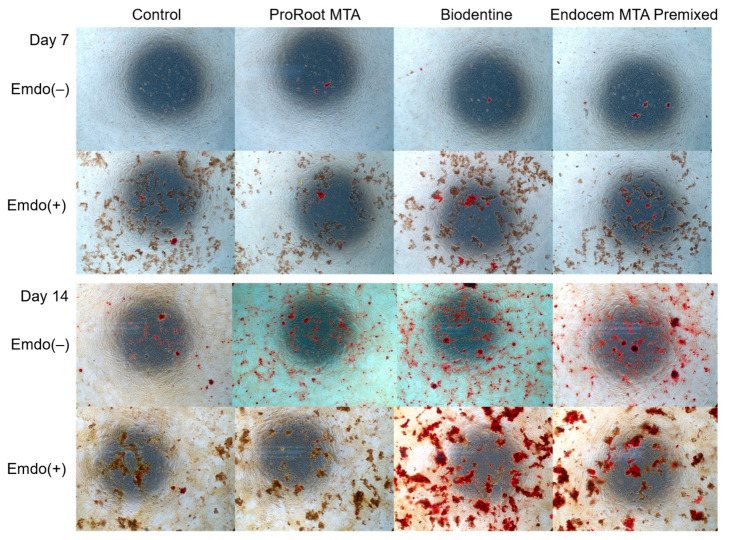
Representative images from ARS staining on Days 7 and 14.

**Figure 7:**
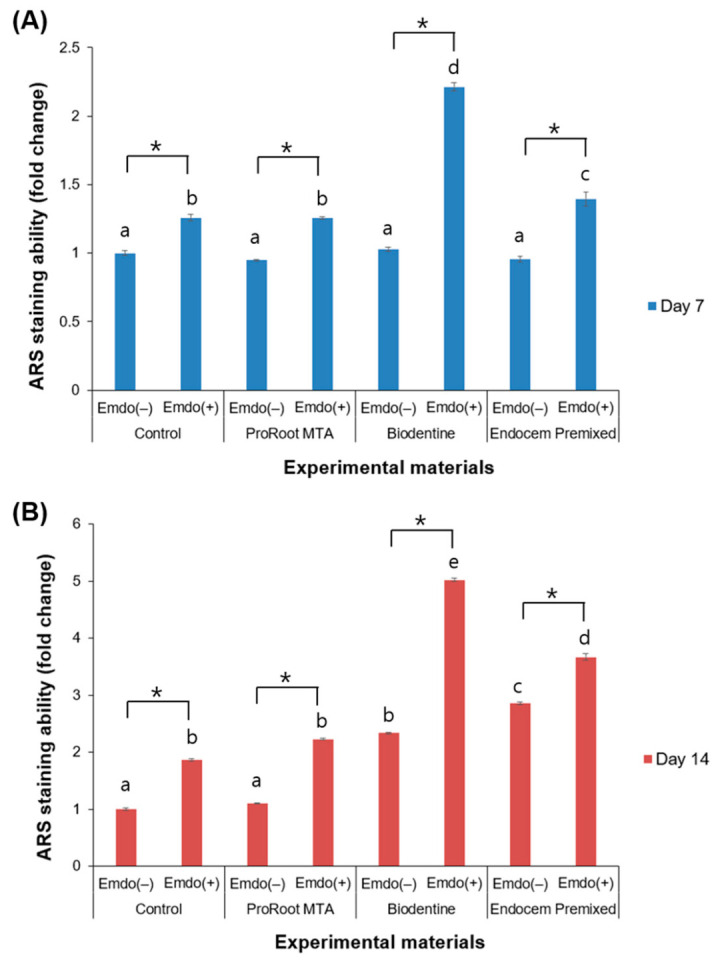
Comparison of ARS staining by the presence or absence of Emdogain gel (Emdo). (**A**) Day 7 and (**B**) Day 14. The different letters indicate significant differences between all groups (*p* < 0.05). The asterisks indicate significant differences between the presence and absence of Emdogain. The error bars indicate standard deviations.

Images from each group are provided in Figure 6. The red-stained area indicates the accumulation of calcium, which increased from Day 7 to Day 14. All of the experimental groups had a significantly increased red-stained area in the presence of the Emdogain gel. When the Emdogain gel was not applied, the experimental and control groups had similar ARS staining results on Day 7 (Figure 7A, *p* > 0.05). However, on Day 14, the Biodentine and Endocem MTA Premixed groups had significantly greater ARS staining than the ProRoot MTA group among the groups without Emdogain (Figure 7B, *p* < 0.05). When the Emdogain gel was applied, all of the experimental groups had significantly higher ARS staining on Days 7 and 14 compared with their counterparts without Emdogain (Figure 7A,B, *p* < 0.05). The results were the highest for the Biodentine group, especially in the presence of Emdogain.

## 4. Discussion

Compared to mature permanent teeth, immature permanent teeth have a relatively large canal space with stronger immune defense due to a high nutritional supply and cell activity. For these reasons, immature permanent teeth are more responsive to endodontic treatment, and successful treatment outcomes are more likely compared to permanent teeth undergoing the same procedure [20]. However, microleakage and clinical failure risks are higher with immature permanent teeth because of the presence of large dentinal tubules, thin root dentin walls, and short roots. MTA, and newly developed HCSCs, are widely used as effective materials for regenerative endodontic procedures in these teeth. In this study, we compared the osteogenic and dentinogenic capabilities of hDPSCs when treated with either ProRoot MTA, Biodentine, or Endocem MTA Premixed, with or without Emdogain gel.

MTT analysis and live/dead staining analysis were used to determine the cell proliferation induced by ProRoot MTA, Biodentine, and Endocem MTA Premixed. MTT analysis is common and standard for evaluating cell viability and indicates the influence of experimental compounds on cell proliferation and cytotoxicity [21,22,23]. Moreover, live/dead staining analysis, which measures the intercellular esterase activity and plasma membrane loss, is an indicator of cell viability. In the MTT assay, all of the experimental groups that were treated with Emdogain gel had significantly higher cell proliferation than those that were not treated with Emdogain. Furthermore, in the live/dead analysis, the green-staining density increased, and the fluorescence intensity was greater in the Emdogain-treated groups. These findings suggest that MTA and HCSCs are biocompatible and that the addition of Emdogain gel enhanced the degree of cell viability via a synergistic effect.

Regarding pulp therapy in immature permanent teeth, the ideal treatment outcome is the regeneration of the dentin–pulp complex; however, the histological properties of newly formed tissue are currently unknown. It has been reported that even in cases with a thickening of the canal wall and continual root growth, newly formed tissue is osteoid-like [24]. In this study, we used ALP analysis, qRT–PCR, and ARS staining to assess the bone formation capacity in the experimental groups.

ALP, an extracellular enzyme that attaches to the outside of the cell membrane, is used as a marker to distinguish cells. There are various types of ALP isotopes, and 95% of all ALPs are formed from the bone and liver. With the induction of osteoblast differentiation, ALP expression increases, making ALP suitable as an early marker of osteoblast differentiation [25]. In the current study, the ALP activity significantly increased on Days 2 and 4 under Emdogain application. However, the groups that were treated with Emdogain versus those that were not did not differ on Day 6. This pattern implies that, initially, most of the hDPSCs differentiated into osteoblasts with MTA and HCSCs, so that, at a later stage, Emdogain had no significant effect on the ALP expression. In addition, on Day 6, ProRoot MTA and Endocem MTA Premixed had higher ALP activity than the control group without the Emdogain gel. ProRoot MTA produces calcium hydroxide and releases calcium ions during the hydration reaction, resulting in high pH and mineralization, which leads to high ALP activation [26,27]. According to its manufacturer, the major component of Endocem MTA Premixed is zirconium dioxide, which accounts for approximately 45–55% of its composition. The inclusion of metal oxides enhances the substance’s mineralization activity [28]. Zirconium naturally occurs in bones and is a biocompatible material that can induce the formation of bone tissue. It can also strengthen the growth and specialization of osteoblast, the cells that are responsible for creating new bone [28].

To analyze the expression levels of the bone formation markers (*RUNX2* and *OSX*) and the odontogenic markers (*DMP-1* and *DSPP*) in each experimental group, we used qRT–PCR, according to previous reports [19,29]. DMP-1 is a non-collagenous protein that is involved in the calcification of hard tissue [30]. It has been described in secondary dentin close to the predentin layer and in the early stages of odontoblast differentiation, when the primary dentin is formed [31]. DSPP is a non-collagenous protein that is found in the predentin layer [32] and in fully polarized dentin in the late differentiation stage [33]. *RUNX2* and *OSX* are early markers of osteoblast differentiation [34,35], and *RUNX2* is also expressed in preodontoblasts and is involved in tooth formation and mineralization [36,37]. The main purpose of vital pulp therapy is to preserve the activity and function of the residual pulp tissue, and thus continue the physiological development of the root. Therefore, the materials used in pulp capping therapy should induce the hDPSCs to differentiate into osteogenic and odontogenic cells, enabling dentin–pulp complex formation and regenerative dentin formation [38,39]. According to the current results, *DSPP* expression on Day 7 in the control, Biodentine, and Endocem MTA Premixed groups significantly increased with the presence of Emdogain gel. The expression levels of *RUNX2* and *OSX* also significantly increased when Emdogain gel was added to the control and Endocem MTA Premixed groups. In the case of ProRoot, the presence or absence of Emdogain did not affect the expression levels of these markers. The findings suggest that Endocem MTA Premixed with Emdogain gel could enhance the differentiation of hDPSCs into osteogenic and odontogenic cells.

According to previous work, amelogenin, the main component of Emdogain, regulates cell signaling pathways by activating Wnt/β-catenin signaling [40]. Pluripotent Oct4 and Sox2 factors show higher expression levels in the apical papilla and can enhance pulp regeneration in the presence of this protein [40]. Other findings indicate that amelogenin exon 5 peptide promotes the expression of MAPK signaling pathway proteins, with enhanced cell proliferation and osteogenic differentiation [41]. The results of these studies are in agreement with the current findings; however, less is known about how amelogenin might influence DPSC odontoblastic differentiation and possible molecular mechanisms [42].

ARS is used to evaluate the level of bone differentiation by staining the calcium accumulated in differentiated bone cells [43]. In the present study, ARS staining increased over time and showed the same tendency when Emdogain gel was applied. These results indicate that, regardless of whether Emdogain gel was applied, the calcium accumulation from the osteoblasts increased. A significant increase in calcium accumulation was present in all of the experimental groups on Days 7 and 14, particularly with Emdogain treatment.

The results of the ARS staining analysis, with increased calcium accumulation up to Day 14, may appear discordant with those of the ALP analysis, which showed an early effect of Emdogain gel, with a declining influence after Day 6. The effects may represent a sequential pattern. The addition of Emdogain gel to MTA or HCSCs could induce a rapid formation of new hard tissues by increasing the initial ALP expression. As the continuous increase in ARS staining seems to suggest, Emdogain gel then seems to contribute to the sufficient formation of new hard tissue. The treatment goal of regenerative endodontic procedures is to regenerate the pulp–dentin complex with the continuous growth of wall thickness and root length. The three factors that are necessary for tissue regeneration are growth factor, stem cells, and scaffold. Blood clotting provides the growth factors and scaffold, and the apical complex provides the stem cells [1]. EMD, consisting of 90% amelogenin, not only acts as a scaffold, but also controls the expression of growth factors, contributing to tissue regeneration [44].

This study has some limitations. First, we assumed that the MTT assay would evaluate cell viability, however, this assay directly measures cell metabolic activity. Although metabolic activity can be considered an indirect indicator of viability, cells without metabolic activity can still be viable. Other assays will be needed for an accurate assessment of cell viability. Second, we investigated all of the experiments in in vitro circumstances, and could not reproduce direct contact of hDPSCs with the experimental disks or eluates. Therefore, in vivo studies are needed in order to determine the ultimate effect of these materials. Third, the current evidence is not sufficient for any conclusions regarding the superiority of one material over another. The results of osteogenic and dentinogenic differentiation may differ depending on the extraction ratio of the experimental disks. Further study is required for a full understanding of the material properties and best methodologies.

Within the limits of this study, it may be concluded that the newly developed HCSCs showed favorable results in terms of cell toxicity and osteogenic/odontogenic differentiation, similar to the outcomes with ProRoot MTA, and demonstrated beneficial effects when combined with EMD. The application of EMD, which enhances the differentiation of hDPSCs into osteoblasts and odontoblasts, can be considered to ensure a successful prognosis from a tissue engineering perspective. Further research is needed in order to determine the effects on the material properties when MTA, HCSCs, and Emdogain gel are applied in combination.

## Figures and Tables

**Table 1:** Manufacturers and compositions of each material used in this study.

Material	Manufacturer	Composition	Final Setting Time	Batch Number
ProRoot MTA	Dentsply Tulsa Dental Specialties, Tulsa, OK, USA	Portland cement (tricalcium silicate, dicalcium silicate, and tricalcium aluminate) 75%	261 min	0000176943
		Calcium sulfate dehydrate (gypsum) 5%		
		Bismuth oxide 20%		
Biodentine	Septodont, Saint-Maur-des-Fosses Cedex, France	Tricalcium silicate 80.1%	12 min	B24553
		Calcium carbonate 14.9%		
		Zirconium oxide 5%		
		Calcium chloride		
		Soluble polymer as an aqueous liquid		
Endocem MTA Premixed	Maruchi, Wonju, Republic of Korea	Zirconium dioxide 45–55%	27 min	FS200831A
		Calcium silicate 20–25%		
		Calcium aluminate 1–5%		
		Calcium sulfate 1–5%		
		Dimethyl sulfoxide 20–25%		
		Thickening agent 1–5%		
Emdogain gel	Straumann, Basel, Switzerland	Amelogenin 90%		FMN92
		Remaining percentage is proline-rich non-amelogenin, tuftelins, tuft proteins, ameloblastin, amelins		

**Table 2:** Primers and sequences used in qRT–PCR.

Runt-related transcription factor 2 (*RUNX2*)	Forward: 5′-AAG TGC GGT GCA AAC TTT CT-3′
	Reverse: 5′-TCT CGG TGG CTG CTA GTG A-3
Osterix (*OSX*)	Forward: 5′-AGC CTC TGG CTA TGC AAA TGA-3′
	Reverse: 5′-TGT AGA CAC TAG GCA GGC AGT CA-3
Dentin matrix protein-1 (*DMP-1*)	Forward: TGG TCC CAG CAG TGA GTC CA
	Reverse: TGT GTG CGA GCT GTC CTC CT
Dentin sialophosphoprotein (*DSPP*)	Forward: 5′-GGG AAT ATT GAG GGC TGG AA-3′
	Reverse: 5′-TCA TTG TGA CCT GCA TCG CC-3 ′
*GAPDH*	Forward: GAA GGT GAA GGT CGG AGT C
	Reverse: GAG ATG GTG ATG GGA TTT C

## Data Availability

The datasets used and/or analyzed during the current study are available from the corresponding author on reasonable request.
